# Asymptomatic Bilateral Demodicosis of the Nipple

**DOI:** 10.7759/cureus.107275

**Published:** 2026-04-18

**Authors:** Chalent Alexakis, Evgenia Kalogridaki, Annezo Marinatou, Aikaterini-Xakousti Maritsa, Panagiotis Daskalakis

**Affiliations:** 1 Rural Medicine Department, General Hospital of Thebes, Thebes, GRC; 2 Surgery Department, Breast Unit, General Maternal Hospital of Athens "Elena Venizelou", Athens, GRC; 3 Obstetrics and Gynecology Department, General Maternal Hospital of Athens "Elena Venizelou", Athens, GRC

**Keywords:** asymptomatic, benign breast condition, breast lesions, demodex, parasite

## Abstract

*Demodex *parasites are obligate ectoparasites found in the sebaceous glands and hair follicles, primarily on the human head and face. They are the largest and most complex organisms in the skin microflora. Their pathogenic role as a cause of dermatoses in humans raises ongoing concerns. The species of *Demodex *mites have been found in skin lesions, but *Demodex *disease has not been accepted as a distinct entity. All skin conditions caused by *Demodex *mites are collectively referred to as demodicosis. This case is an incidental finding of bilateral nipple demodicosis in an otherwise healthy patient who presented to our breast unit for the first time for her annual screening mammogram. It highlights an unusual location of *Demodex *infestation, with bilateral nipple involvement, a finding that appears to be rare in the existing literature. Increased awareness may lead to earlier recognition and appropriate management.

## Introduction

The term "human demodicosis" refers to skin conditions caused by the presence of two species of *Demodex*: *Demodex folliculorum *and *Demodex brevis*, which affect the sebaceous glands and hair follicles, primarily on the scalp and face [1]. In particular, *Demodex* mites are part of the normal cutaneous flora and are frequently present in low numbers in healthy individuals without causing symptoms. Human *Demodex *mites (*D. folliculorum *and *D. brevis*) are the largest and most complex organisms in the skin's flora. The pathogenesis of demodicosis and the human immune system's response to mite invasion are not yet fully understood [2].

The correct classification of these mites was made in 1842 by dermatologist Carl Gustav Theodor Simon for *D. folliculorum *[3], and *D. brevis *was identified by Akbulatova in 1963 [4]. Of the two species parasitizing humans, *D. folliculorum *is usually found in hair follicles, while the smaller *D. brevis *affects the sebaceous glands [5].

The presence of *Demodex *species in low concentrations on healthy skin is common and typically remains asymptomatic [6]. In contrast, high concentrations of these mites have been observed on the skin of patients with various dermatological conditions, including papulopustular rosacea, follicular pityriasis, and perioral dermatitis, as well as in patients with immunosuppression due to diseases or immunosuppressive drugs [7,8].

Even though *Demodex *infestation most frequently affects sebaceous-rich areas, such as the face, involvement of other anatomical sites is extremely rare. In particular, nipple involvement - especially bilateral involvement - has been occasionally reported in the literature, representing a gap in current clinical knowledge. This rarity may lead to under-recognition and misdiagnosis in clinical practice [9].

## Case presentation

**Figure 1:**
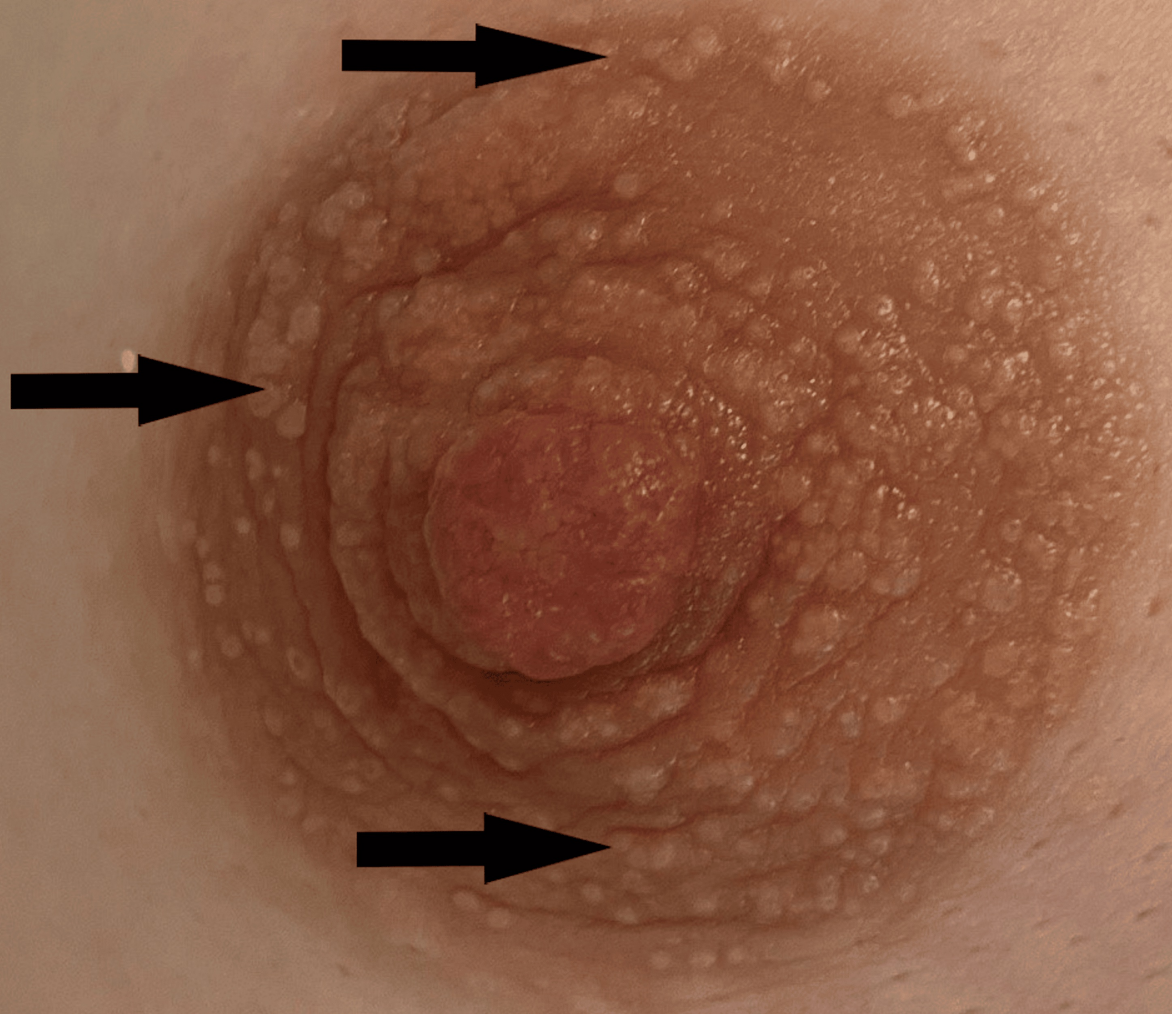
Arrows pointing to the scaling and erythema of the areola of the nipple.

A 45-year-old female, a non-smoker, presented to our breast unit for the first time for her annual screening with a mammogram and breast ultrasound. According to her medical history, the patient was in good health and not taking any medications (including immunosuppressive drugs or corticosteroids). She had no known allergies. The imaging tests revealed dense breast tissue with no changes from the previous mammogram and no evidence of masses (Breast Imaging Reporting and Data System Category 2, BIRADS 2). On clinical examination, no palpable masses were detected, but erythema and scaling were noted around the areola of both nipples, though the patient reported no pruritus or discomfort. Upon further questioning, the patient reported that these changes had been present since adolescence. The rest of the breast tissue appeared normal (Figure 1).

The initial presentation prompted consideration of several differential diagnoses, including contact dermatitis, eczema, and infectious causes, such as bilateral candidal infection, given their relatively common occurrence and similar clinical appearance. Paget's disease of the breast was also considered due to its potential to present with nipple erythema, crusting, and scaling and its association with underlying malignancy. However, the absence of a palpable breast mass and the absence of imaging findings made this diagnosis unlikely. Ultimately, a full-thickness skin biopsy was recommended, and with the patient's consent, a small skin incision was made under local anesthesia.

**Figure 2:**
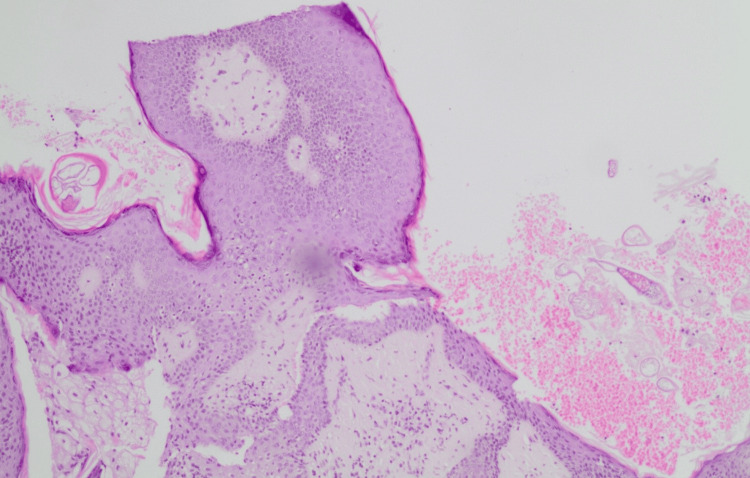
Histological section of nipple tissue stained with hematoxylin and eosin (H&E), highlighting a parasitic infection. The central feature is a cross-section of a parasitic mite encased in a cyst-like structure within the tissue, surrounded by layers of epithelial cells (original magnification ×10).

**Figure 3:**
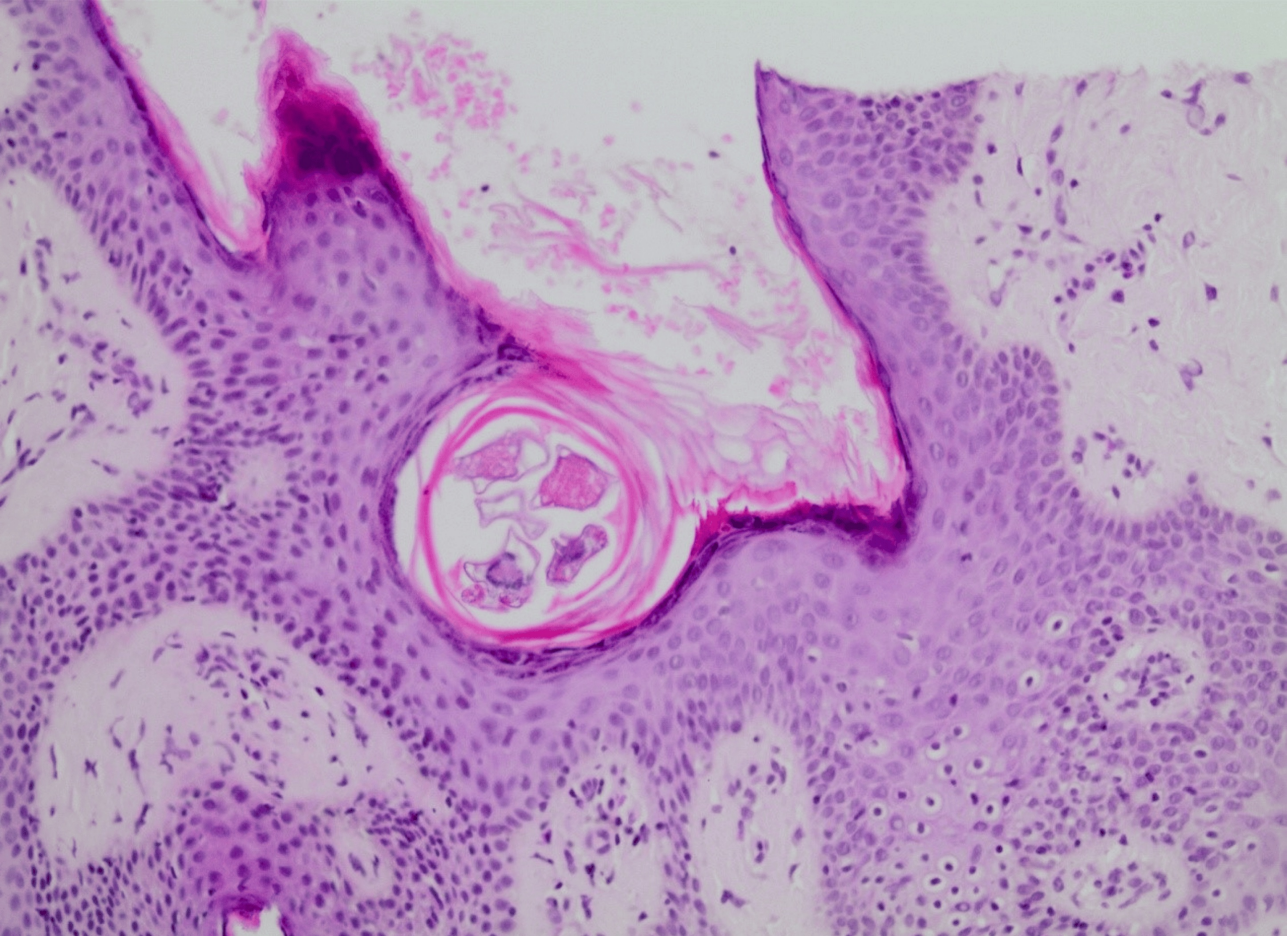
Cross-section of the nipple tissue stained with H&E, displaying a parasitic mite embedded within the host's epidermis, alongside evidence of hemorrhage in the nearby region. The mite is housed within a circular cyst-like structure in the left area of the image, while the right portion shows a large, pale pink area indicative of blood cells and possible tissue damage. The surrounding tissue shows dense cellular organization, with inflammation and immune response visible (original magnification ×10).

The specimen was sent for histopathological analysis. Upon examination, the histopathologist concluded that a section of nipple areola skin exhibits significant lymphoplasmacytic and histiocytic inflammation in the papillary dermis, with the formation of incomplete granulomas. The inflammatory cells are primarily arranged around adnexal structures and blood vessels and extend into the overlying epidermis in some areas. The epidermis shows acanthosis and hyperkeratosis. *Demodex *mites are identified in both the keratin layer and the openings of hair follicles and sebaceous glands. These findings are consistent with *Demodex *dermatitis (demodicosis) (Figures 2-3). This correlates with the patients' clinical presentation of persistent scaling and chronic erythema of both nipples.

Administration of 1% ivermectin cream twice a day with 10% urea led to complete remission of the skin lesions after four weeks of treatment. Topical ivermectin was selected as it is currently among the most evidence-supported topical agents targeting *Demodex *mites. The patient did not show any signs of recurrence after a three-month follow-up.

## Discussion

The clinical form of demodicosis that manifests in each individual depends on the degree of infestation by *Demodex *mites, the duration of the disease, the patient's age, and their overall health condition [6]. The term "human demodicosis" refers to a skin disease affecting the sebaceous glands and hair follicles, primarily on the scalp and face, and is associated with the presence of the two species of *Demodex *that infect humans: *D. folliculorum *and *D. brevis *[1].

The diagnostic criteria for primary demodicosis include the following: (a) the absence of pre-existing or concurrent inflammatory dermatoses such as acne, rosacea, or perioral dermatitis; (b) detection of an unusual increase in *Demodex *mite colonization in skin lesions after direct examination; and (c) resolution of symptoms following appropriate topical or systemic treatment targeting mites, as opposed to antibiotics such as macrolides or tetracyclines [1,10].

*Demodex *mites have a limited life cycle outside the human body due to their vulnerability to dehydration. Direct contact is necessary for transmission between individuals. Therefore, effective treatment for demodicosis must target both the eradication of mites and the prevention of their reproduction and transmission [11].

The treatment of human demodicosis is primarily based on individual case reports. Factors that complicate treatment selection include the inability to culture *Demodex *species, which makes it difficult to assess the effectiveness of pharmaceutical agents against the parasite. Furthermore, distinguishing between primary demodicosis and inflammatory diseases such as rosacea, with or without secondary demodicosis, adds diagnostic complexity. Many pharmaceutical agents have dual actions, including anti-inflammatory and antimicrobial effects [2].

The present case contributes to the expanding clinical spectrum of demodicosis by highlighting an unusual anatomical localization. Involvement of the nipple is extremely rare and infrequently described in the existing literature. Fewer than 20 cases have been described since 1946, most featuring unilateral disease or symptomatic presentations (e.g., erosion, sinus formation). Bilateral involvement has been rarely reported in the literature, with most published cases describing unilateral or localized disease [9]. This atypical presentation raises important diagnostic considerations, particularly in differentiating demodicosis from other inflammatory, infectious, or dermatologic conditions that may affect specialized cutaneous regions.

## Conclusions

Human demodicosis manifests as the asymptomatic infestation of humans by the two species of *Demodex *mites: *D. folliculorum *and *D. brevis*. The role of these mites in demodicosis should be considered, as they can present with a variety of clinical manifestations that mimic other dermatological conditions. The existence of sebaceous glands in the nipple and areola establishes a suitable microenvironment for colonization by those mites. However, involvement of this anatomical site is exceedingly rare and infrequently reported in the literature. It is an underdiagnosed clinical entity, and if suspicion is high, a definite diagnosis should be made by histopathological examination. Increased awareness can lead to timely diagnosis and appropriate treatment in order to achieve complete resolution of symptoms. Bilateral nipple involvement, as seen in this case, is uncommon and has rarely been reported in the literature. Further studies are required to better understand the epidemiology and clinical significance of demodicosis in this specific anatomical region.
